# Newly Detected Atrial Flutter After Enclomiphene Dose Escalation in a Patient With Prior Mitral Valve Repair and Chronic Immune Thrombocytopenia: A Case Report

**DOI:** 10.7759/cureus.114562

**Published:** 2026-08-15

**Authors:** Muhammad N Tariq, Ahmed T Abdel Halim, Jennifer A Evans

**Affiliations:** 1 Medicine, Ayub Medical College, Abbottabad, PAK; 2 Internal Medicine, MedStar Franklin Square Medical Center, Baltimore, USA; 3 Internal Medicine, The Hospital of Central Connecticut, New Britain, USA

**Keywords:** adult atrial flutter, adult congenital heart disease, drug-induced arrhythmia, enclomiphene citrate, role of testosterone

## Abstract

Enclomiphene citrate is increasingly prescribed off-label for secondary male hypogonadism, and its cardiovascular safety profile remains poorly characterised.

We report a 46-year-old man with a mitral valve repair performed 20 years earlier and chronic immune thrombocytopenia (ITP) in whom atrial flutter was first documented shortly after he inadvertently increased his enclomiphene dose, taking 50 mg daily in place of 25 mg daily. Laboratory evaluation demonstrated chronic thrombocytopenia with a platelet count of 36 × 10⁹/L, while thyroid function, cardiac biomarkers, and electrolytes were normal. Transthoracic echocardiography showed preserved left ventricular systolic function with mild asymmetric left ventricular hypertrophy, mild left atrial enlargement, and mildly reduced right ventricular systolic function. Computed tomography pulmonary angiography (CTPA) excluded pulmonary embolism. Rate control was achieved with metoprolol and diltiazem. Anticoagulation was deferred because of severe thrombocytopenia, and high-dose prednisone was initiated to raise the platelet count before planned anticoagulation and elective cardioversion.

Testosterone was elevated at presentation and the arrhythmia was detected soon after the dose increase, but causality cannot be established. The patient had a remote atriotomy and echocardiographic evidence of chronic atrial and ventricular remodelling, a substrate sufficient on its own to sustain macro-reentrant atrial flutter, and earlier asymptomatic or self-limiting episodes could not be excluded. Formal causality assessment using the Naranjo scale placed the association in the possible category only. The case is presented for two reasons: as an illustration of how a hormonal agent should be weighed against a pre-existing structural substrate rather than assumed to be causal, and as an example of the difficulty of balancing thromboembolic and bleeding risk when atrial flutter coexists with severe ITP.

## Introduction

Enclomiphene citrate is the trans-isomer of clomiphene citrate, a non-steroidal oestrogen receptor antagonist used off-label for many years in the treatment of secondary male hypogonadism, particularly in the setting of male infertility. Unlike exogenous testosterone, it increases gonadotropin release, which results in increased testicular stimulation and endogenous testosterone production [1]. Its cardiovascular safety profile remains poorly studied, and population data have linked higher endogenous testosterone levels with incident atrial arrhythmias in men [2].

Nonetheless, attributing a newly documented arrhythmia to a recently escalated drug is difficult when the patient already carries a strong anatomical substrate. Macro-reentrant atrial tachycardias are a recognised late consequence of mitral valve surgery, and patients with chronic immune thrombocytopenic purpura (ITP) present the additional problem that bleeding risk constrains the anticoagulation required for rhythm control.

We describe a patient in whom atrial flutter was first documented shortly after enclomiphene dose escalation. We use the case to examine how a possible hormonal contributor should be weighed against a pre-existing post-surgical atrial substrate, and how anticoagulation and rhythm control are managed when severe thrombocytopenia constrains both.

## Case presentation

A 46-year-old male with a remote history of mitral valve prolapse, status post-surgical repair 20 years earlier, and ITP was admitted with progressive exertional dyspnoea and fatigue. These symptoms developed shortly after the patient inadvertently increased his enclomiphene dose from 25 mg to 50 mg daily. He denied chest pain, palpitations, syncope, fever, cough, or urinary symptoms. There was no known history of coronary artery disease, hypertension, diabetes mellitus, or chronic kidney disease. He had no history of tobacco or alcohol use. His family history was significant for sudden cardiac death. Social determinants of health included social isolation, transportation limitations, and financial strain.

**Figure 1:**
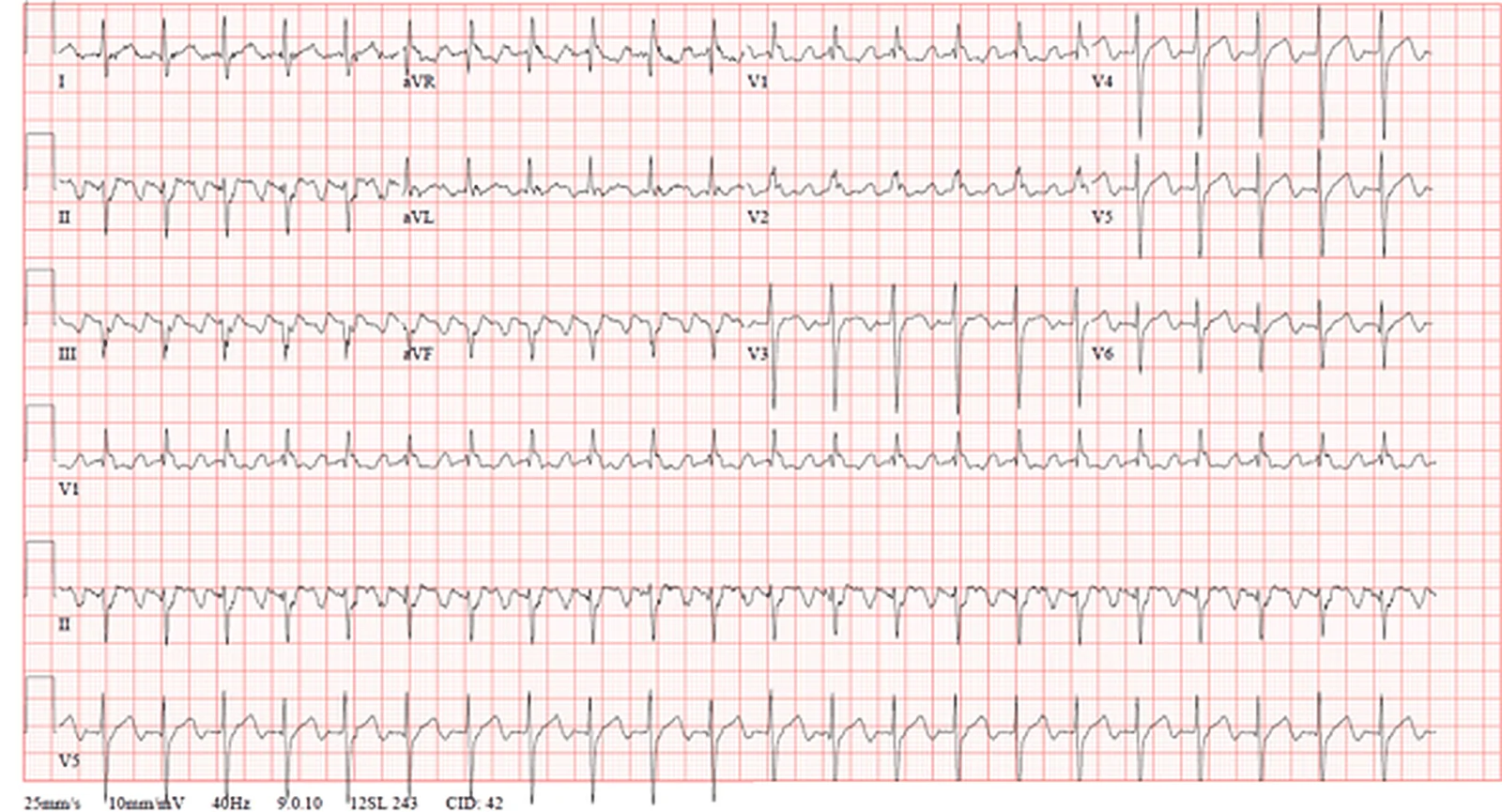
Admission electrocardiogram demonstrating atrial flutter

An admission electrocardiogram demonstrated atrial flutter with 2:1 atrioventricular conduction and a ventricular rate of 137 beats per minute (Figure 1). The patient remained haemodynamically stable and was asymptomatic except for exertional dyspnoea. Physical examination revealed no signs of respiratory distress. Cardiac auscultation demonstrated a regular rhythm without murmurs, rubs, or gallops. Pulmonary, abdominal, neurological, and extremity examinations were unremarkable.

Initial laboratory evaluation revealed thrombocytopenia with a platelet count of 36 × 10⁹/L. N-terminal proB-type natriuretic peptide (NT-proBNP) was within the normal range, and troponin was negative. Thyroid function tests and serum electrolytes were normal. Review of previous laboratory records showed persistent thrombocytopenia, with a platelet count of 43 × 10⁹/L one month earlier, suggesting a chronic underlying process. The remaining laboratory investigations, including white blood cell count, basic metabolic panel, and coagulation profile, were within normal limits. Testosterone levels were elevated at 1400 ng/dL. A summary of the key laboratory and diagnostic findings is provided in Table 1.

**Table 1:** Laboratory and diagnostic findings LV, left ventricle or left ventricular; RV, right ventricle or right ventricular; NT-proBNP, N-terminal pro-B-type natriuretic peptide; CTPA, computed tomography pulmonary angiography

Parameter	Result	Reference range
Platelet count on admission	36 × 10⁹/L	150 to 400 × 10⁹/L
Previous platelet count	43 × 10⁹/L (one month prior)	150 to 400 × 10⁹/L
White blood cell count	Within normal limits	4.0 to 11.0 × 10⁹/L
Basic metabolic panel	Within normal limits	Sodium: 135 to 145 mmol/L, Potassium: 3.5 to 5.0 mmol/L, Chloride: 98 to 107 mmol/L, Bicarbonate: 22 to 29 mmol/L, Urea: 2.5 to 7.8 mmol/L, Creatinine: approximately 45 to 90 μmol/L (female). Adjust according to local laboratory reference ranges.
Coagulation profile	Within normal limits	Prothrombin time: 11 to 13.5 s, International normalized ratio: 0.8 to 1.2, Activated partial thromboplastin time: 25 to 35 s. Adjust according to local laboratory reference ranges.
NT-proBNP	Normal	<125 pg/mL (patients <75 years), or according to local laboratory reference range
Troponin	Negative	Below the assay-specific 99th percentile upper reference limit
Thyroid function tests	Normal	Thyroid-stimulating hormone: 0.4 to 4.0 mIU/L, Free thyroxine: 10 to 23 pmol/L
Transthoracic echocardiography	Normal left ventricular systolic function, mild asymmetric left ventricular hypertrophy, mild left atrial enlargement, mildly reduced right ventricular systolic function	N/A
Testosterone at presentation	1400 ng/dL	Adult males: 265-923 ng/dL

Transthoracic echocardiography demonstrated preserved left ventricular systolic function, mild asymmetric left ventricular hypertrophy, mild left atrial enlargement, and mildly reduced right ventricular systolic function. Mild regurgitation was observed across all four cardiac valves. Computed tomography pulmonary angiography (CTPA) excluded pulmonary embolism, and chest radiography showed no acute cardiopulmonary abnormalities.

The differential diagnosis for exertional dyspnoea and newly identified atrial flutter included enclomiphene-associated arrhythmia, structural heart disease related to previous mitral valve repair, ischemic heart disease, thyrotoxicosis, and pulmonary embolism. Ischemic heart disease was considered less likely due to the absence of significant cardiovascular risk factors, negative troponin results, and lack of supportive clinical findings. Thyrotoxicosis was excluded based on normal thyroid function tests, and pulmonary embolism was ruled out by CTPA.

Given that the duration of atrial flutter exceeded three weeks, cardioversion required anticoagulation before and after the procedure to reduce thromboembolic risk. However, anticoagulation could not be initiated immediately because of severe thrombocytopenia. The patient was therefore scheduled for transoesophageal echocardiography (TEE)-guided outpatient cardioversion once anticoagulation could be safely established.

Haematology consultation was obtained. Due to the absence of specific clinical guidelines addressing anticoagulation in patients with ITP, a platelet threshold-based strategy was adopted. Anticoagulation was recommended once platelet counts exceeded 50 × 10⁹/L. The patient was started on oral prednisone 100 mg daily, followed by a planned 10-week taper, reducing the dose by 10 mg each week. This approach was selected to achieve a sustained improvement in platelet count and facilitate safe initiation and continuation of anticoagulation for cardioversion.

For ventricular rate control, the patient was started on metoprolol tartrate 50 mg twice daily and diltiazem 240 mg daily. The therapeutic interventions and their clinical rationale are summarised in Table 2.

**Table 2:** Therapeutic interventions and clinical rationale

Intervention	Details	Clinical rationale
Corticosteroid therapy	Prednisone 100 mg daily followed by taper of 10 mg weekly over 10 weeks	To increase platelet count in suspected immune thrombocytopenia and facilitate safe anticoagulation
Anticoagulation strategy	Planned once platelet count exceeded 50 × 10⁹/L	To minimise bleeding risk while allowing thromboembolic protection during atrial flutter management
Rate control therapy	Metoprolol tartrate 50 mg twice daily and diltiazem 240 mg daily	To achieve ventricular rate control
Rhythm control strategy	Transoesophageal echocardiography (TEE)-guided outpatient cardioversion	Deferred until anticoagulation could be safely initiated and maintained

The patient remained haemodynamically stable throughout hospitalisation and showed no clinical evidence of heart failure. He was discharged with close follow-up under cardiology and haematology services, continuation of the prednisone taper, and ongoing rate control therapy. Cardioversion was deferred until platelet recovery allowed safe initiation and maintenance of anticoagulation. Rhythm and hormonal outcomes after discharge were not available at the time of reporting.

## Discussion

Surgical repair of the mitral valve creates a durable arrhythmogenic substrate. Atriotomy and cannulation scars, suture lines, and the conduction barriers they impose provide fixed obstacles around which macro-reentrant circuits organise, and atrial tachyarrhythmias are a well-described late outcome of mitral valve surgery [3-5]. In a series of 67 patients presenting with atrial tachycardias after mitral valve surgery, macro-reentry was the predominant mechanism, with typical cavotricuspid isthmus-dependent flutter the single most common circuit and incisional right atrial and perimitral circuits also frequent [3]. The incidence and mechanism depend heavily on the surgical access route, which determines where the scar lies [4,5]. Our patient's echocardiogram demonstrated left atrial enlargement, asymmetric left ventricular hypertrophy, mildly reduced right ventricular systolic function, and mild regurgitation across all four valves, findings that reflect chronic structural change rather than an acute process and that correspond to the concept of atrial cardiomyopathy as a substrate for atrial arrhythmia [6]. Chronic ITP may add an inflammatory contribution to atrial remodelling [7]. Taken together, this post-surgical and remodelled substrate can be a predisposing factor for arrhythmia in this patient. However, development of symptoms only after enclomiphene may suggest the drug to be a secondary modulator acting on a heart already predisposed to flutter.

The onset of the arrhythmia could not be dated. The patient was asymptomatic apart from exertional dyspnoea and denied palpitations, and the estimated flutter duration already exceeded three weeks at presentation. No prior ambulatory monitoring, wearable rhythm data, or recent electrocardiogram was available. Subclinical or self-limiting episodes of atrial flutter preceding the dose escalation therefore cannot be excluded, and the arrhythmia is more accurately described as newly detected than as new in onset. Left atrial enlargement at presentation is itself consistent with a longer-standing atrial process. In the absence of electrophysiological study and electroanatomical mapping, the circuit was not characterised, and it was not possible to determine whether the flutter was cavotricuspid isthmus-dependent or scar-related, a distinction that would have carried direct implications for how much weight the surgical history should bear.

A hormonal contribution nonetheless remains biologically plausible. Testosterone influences cardiac electrophysiology and clinical cardiac outcomes in men [8], and in the UK Biobank, higher calculated free testosterone was associated with an increased risk of incident atrial fibrillation and ventricular arrhythmias, with sex hormone-binding globulin independently associated with arrhythmia risk [2]. Although enclomiphene does not prolong the QT interval, clomiphene blocks the human ether-à-go-go-related gene (hERG) potassium channel, which can alter atrial electrophysiology and may facilitate re-entry in an atrium that already has substrate abnormalities [9]. Drug-induced atrial arrhythmia is a recognised clinical entity involving several drug classes, including anti-oestrogens, through direct electrophysiological effects, autonomic modulation, and pro-inflammatory mechanisms [10]. Plausibility, however, is not evidence of causation in an individual patient.

To make the strength of the association explicit rather than rhetorical, the Naranjo adverse drug reaction probability scale was applied [11]. The event followed the dose increase and was confirmed by objective electrocardiographic evidence, and it coincided with a doubling of the dose, but a plausible alternative cause was present, and neither dechallenge nor rechallenge data were available. The resulting score placed the association in the possible category, not the probable category. The item-level assessment is shown in Table 3. We report this score to define the limits of what the case can support.

**Table 3:** Naranjo adverse drug reaction probability assessment for the temporal association between enclomiphene dose escalation and atrial flutter Interpretation: total score 9 or more, definite; 5 to 8, probable; 1 to 4, possible; 0 or less, doubtful. Scale reference: [11]

Item	Naranjo scale question	Score	Basis in this case
1	Are there previous conclusive reports on this reaction?	0	No previously published reports of enclomiphene-associated atrial flutter were identified.
2	Did the adverse event appear after the suspected drug was administered?	2	Exertional dyspnoea began after the inadvertent dose increase to 50 mg daily, and atrial flutter was documented on the admission electrocardiogram.
3	Did the adverse event improve when the drug was discontinued or a specific antagonist was administered?	0	No dechallenge data available at the time of reporting.
4	Did the adverse event reappear when the drug was readministered?	0	Rechallenge was not performed and was not clinically appropriate.
5	Are there alternative causes that could on their own have caused the reaction?	-1	Mitral valve repair 20 years earlier with atriotomy scarring, left atrial enlargement, mild asymmetric left ventricular hypertrophy, and mildly reduced right ventricular systolic function provide an independent arrhythmogenic substrate.
6	Did the reaction reappear when a placebo was given?	0	Not applicable, No placebo was administered.
7	Was the drug detected in blood or other fluids in concentrations known to be toxic?	0	No enclomiphene concentration was measured, and no toxic threshold is established. Serum testosterone was elevated at 1400 ng/dL but is a pharmacodynamic marker, not a drug level.
8	Was the reaction more severe when the dose was increased, or less severe when the dose was decreased?	1	The arrhythmia was detected after the daily dose was doubled. No dose reduction data are available.
9	Did the patient have a similar reaction to the same or similar drugs in any previous exposure?	0	No previous exposure to enclomiphene at a higher dose or to related selective oestrogen receptor modulators.
10	Was the adverse event confirmed by any objective evidence?	1	Twelve-lead electrocardiogram documenting atrial flutter with 2:1 atrioventricular conduction at a ventricular rate of 137 beats per minute.
	Total score	3	Possible adverse drug reaction

The second element of the case is management. Thrombocytopenia constrains anticoagulation and therefore constrains the timing and coordination of rhythm control. Current guidelines recommend risk-based anticoagulation for atrial flutter [12,13], but severe thrombocytopenia complicates that recommendation because bleeding risk rises as platelet counts fall. A platelet threshold-based strategy, as used here, is consistent with the approach reported in patients with severe thrombocytopenia and atrial arrhythmias, in whom a count above 50 × 10⁹/L is commonly used as the point at which therapeutic anticoagulation becomes acceptable [14-17]. Corticosteroid therapy served the dual purpose of treating the underlying ITP and creating a window in which anticoagulation and subsequent cardioversion could be undertaken safely.

## Conclusions

In this patient, atrial flutter was first documented after enclomiphene dose escalation, but a mitral valve repair performed 20 years earlier, with the atrial scarring and chronic remodelling that follow it, provides an independent and probably dominant explanation, and earlier asymptomatic episodes could not be excluded. Formal causality assessment supported no more than a possible association. No causal relationship can be inferred from a single case.

The case is instructive in two respects. First, when a new atrial arrhythmia is identified in a patient taking a hormonal agent, the differential should include both the drug and the pre-existing structural substrate, and the substrate should not be overlooked because the drug is the more recent change. Where the distinction matters clinically, electrophysiological study can characterise the circuit and clarify the contribution of surgical scar. Second, atrial flutter occurring alongside severe ITP forces a difficult balance between thromboembolic and bleeding risk, and a platelet threshold-based strategy combined with corticosteroid therapy offers a practical route to safe anticoagulation and eventual cardioversion.
